# Clinical Characteristics and a Novel Prediction Nomogram (EASTAR) for Patients with Hemorrhagic Fever with Renal Syndrome: A Multicenter Retrospective Study

**DOI:** 10.3390/tropicalmed10020051

**Published:** 2025-02-08

**Authors:** Ke Ma, Ting Wu, Wei Guo, Jun Wang, Quan Ming, Jun Zhu, Hongwu Wang, Guang Chen, Xiaojing Wang, Weiming Yan, Xiaoping Luo, Tao Chen, Qin Ning

**Affiliations:** 1 State Key Laboratory for Diagnosis and Treatment of Severe Zoonotic Infectious Diseases, Department and Institute of Infectious Disease, Tongji Hospital, Tongji Medical College, Huazhong University of Science and Technology, 1095, Jiefang Avenue, Wuhan 430030, China; markusleo@hust.edu.cn (K.M.); wuting@tjh.tjmu.edu.cn (T.W.); weig369@aliyun.com (W.G.); hongwuwang@126.com (H.W.); chenguang@tjh.tjmu.edu.cn (G.C.); wxjmoon@hotmail.com (X.W.); ywm_net@tjh.tjmu.edu.cn (W.Y.); qning@vip.sina.com (Q.N.); 2 Department of Infectious Disease, Qianjiang City Central Hospital, 22, Zhanghua Zhong Road, Qianjiang 433100, China; qjn6249223@163.com; 3 Department of Infectious Disease, Yichang City Third People’s Hospital, 23, Gangyao Road, Yichang 443000, China; 17362720960@163.com; 4 Department of Infectious Disease, Xianning City Central Hospital, 228, Jingui Road, Xianning 437000, China; haoyunzhujun@sina.com; 5 Department of Pediatrics, Tongji Hospital, Tongji Medical College, Huazhong University of Science and Technology,1095, Jiefang Avenue, Wuhan 430030, China; xpluo@tjh.tjmu.edu.cn

**Keywords:** hemorrhagic fever with renal syndrome, clinical features, risk score, prognostic model, calibration curve, ROC analysis

## Abstract

Background: The fatality rate of hemorrhagic fever with renal syndrome (HFRS), due to hantavirus transmitted by rodents, ranges from 1% to 12%. This study aims to delineate the clinical and laboratory characteristics of HFRS, identify factors associated with disease severity, and construct and validate a nomogram for prognosis prediction of HFRS in the central part of China. Methods: Out of 598 HFRS patients diagnosed via serology tests from four hospitals in Hubei Province, 551 were included. Clinical data were gathered and analyzed, followed by logistic univariate and multivariate analyses to identify independent prognostic factors. A nomogram was developed and validated to forecast the patient’s prognosis. Results: Vaccination led to a notable drop in HFRS incidence from 2018 to 2019, and seasonal trends exhibited bimodal changes with peaks from May to July and November to January. The 30-day mortality rate was 4.17% (23/551). Red blood cell count (RBC), age, two-stage overlap, qSOFA ≥ 2, aspartate aminotransferase (AST), and three-stage overlap were identified as independent prognostic factors. A predictive risk classification system using a nomogram chart was developed, and Kaplan–Meier curves indicated that the new system accurately distinguished 30-day mortality among the three risk groups. Conclusions: The risk score (EASTAR) system demonstrated good predictive performance for prognostic prediction, and it can be applied to quickly screen patients who require ICU admission.

## 1. Background

Hantaviruses, known to induce hemorrhagic fever with renal syndrome (HFRS, also known as epidemic hemorrhagic fever), nephropathiaepidemica (NE), and hantavirus cardiopulmonary syndrome (HCPS), represent a significant global zoonotic health threat [1]. HFRS is characterized by symptoms including acute fever, headache, myalgia, abdominal pain, osphyalgia, and dorsalgia and can progress to hemorrhage, hypotension, and acute kidney injury [2]. Classic HFRS is divided into five phases: fever stage, hypotensive stage, oliguric stage, polyuric stage, and convalescent stage. The severity of the disease ranges from mild to severe, with mortality rates ranging from 1% to 12%, depending on the specific viral strain [3]. Vascular leakage due to increased capillary permeability is central to the pathogenesis of hantavirus disease. Unfortunately, no specific antiviral medications are targeting hantaviruses currently [4]. The treatment for HFRS primarily involves supportive care measures, encompassing the maintenance of fluid and electrolyte balance. In cases of complications such as acute kidney injury (AKI), fluid overload, pulmonary edema, and electrolyte imbalance, continuous renal replacement therapy (CRRT) is often employed.

Accurate prediction of prognosis is vital to optimizing treatment and improving outcomes of HFRS. ICU-related prediction models, including the Sequential Organ Failure Assessment (SOFA) and quick SOFA (qSOFA) scores, are commonly used to predict the severity of the disease. Although there are a few studies on prognosis prediction models specific to HFRS, no widely accepted scoring system like the Model for End-Stage Liver Disease (MELD) score for end-stage liver disease has been established. Nomograms convert complex regression equations into visual graphs to simplify the prognostic evaluation [5]. Studies have developed nomograms to predict the prognosis of HFRS, such as those by Li [6] and Yang [7]. To explore a novel prediction model with enhanced clinical applicability, we conducted a retrospective, multicenter, continuous cohort study involving large longitudinal samples of HFRS patients in the central part of China, an endemic area for hantavirus transmission. This study aims to describe the clinical and laboratory characteristics of HFRS, identify factors associated with the disease severity, and construct a nomogram predicting the disease prognosis using laboratory data and the qSOFA score collected upon hospital admission.

## 2. Methods

### 2.1. Study Population

**Figure 1:**
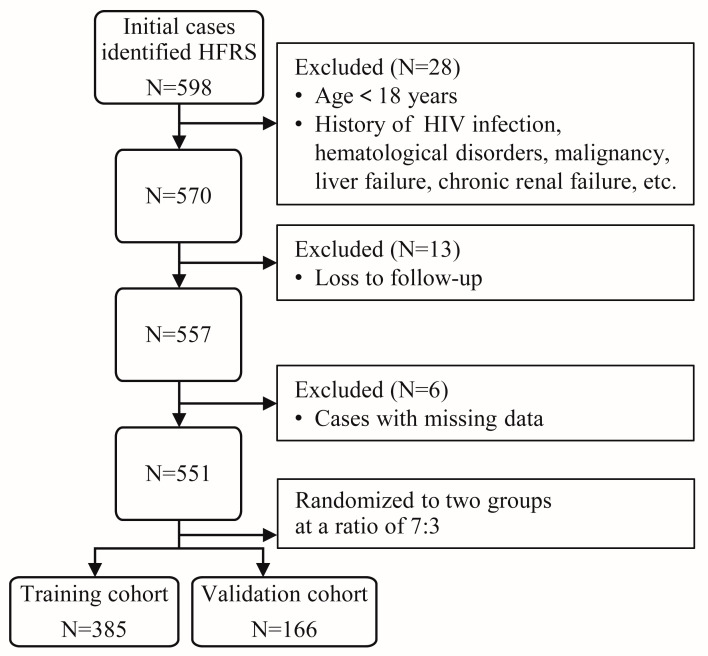
Flowchart of retrospective, multicenter cohort study.

A retrospective, multicenter cohort study was conducted from 1 January 2016 to 31 December 2021, utilizing a study population extracted from Qianjiang City Central Hospital, Tongji Hospital affiliated to Tongji Medical College of Huazhong University of Science and Technology, Yichang City Third People’s Hospital, and Xianning City Central Hospital. HFRS cases were extracted from participating hospitals’ electronic records by searching for patients discharged with “Epidemic Hemorrhagic Fever” or “Hemorrhagic Fever with Renal Syndrome” or using the ICD-10 code. Patients meeting all inclusion and exclusion criteria provided informed consent and were included. A total of 598 patients underwent initial screening. The flowchart illustrating patient enrollment in this study can be seen in Figure 1. This study was conducted following the principles of the Declaration of Helsinki and approved by the local ethics and institutional review boards. Patient’s personal data will not be shared with others to protect their confidentiality.

### 2.2. Inclusion and Exclusion Criteria

All patients gave informed consent prior to enrollment. Inclusion eligibility depended on meeting all inclusion criteria and not meeting any exclusion criteria. Following the exclusion of patients with incomplete data at baseline, none of the enrolled patients had missing clinical information. The inclusion criteria comprised age ≥18 years old and hospitalization with a confirmed diagnosis of HFRS. Patients were required to meet one of the following laboratory results: a positive serum-specific IgM antibody test for hantaviruses or detection of hantaviruses specific IgG antibodies within four days of symptom onset, with an increase in antibody levels of more than four times at an interval of at least one week. The exclusion criteria included superimposed primary chronic kidney disease, known or suspected immunodeficiency (such as human immunodeficiency virus infection, primary immunodeficiency), and mental illness; history of malignancy (including solid malignant tumors and hematological malignancies); recent use of potentially nephrotoxic drugs; current or potential pregnancy; use of immunosuppressive medications (such as glucocorticoids), as well as alcohol addiction and drug abuse.

### 2.3. Clinical Staging of HFRS and qSOFA Score

Diagnosis criteria for patients with HFRS can be referred to published criteria [4]. Fever was defined as an axillary temperature above 37.3 °C. Hypotension was diagnosed as systolic blood pressure of ≤90 mmHg or decreased MAP by greater than 20% from baseline, with increased heart rate, the temperature was lowered in all limbs, lip cyanosis, and skin pallor or mottling. The oliguria patient had a daily urine output below 400 mL. Polyuric was defined as an increase in urine volume from 400 mL to 2000 mL or more per 24 h. The convalescent stage refers to the period after polyuria, when urine volume returned to less than 2000 mL, and other clinical signs and symptoms resolved.

The qSOFA score is calculated based on three criteria: ① systolic blood pressure ≤ 100 mmHg, ② respiratory rate ≥ 22 breaths per minute, and ③ altered mentation [8]. The presence of any of these conditions is assigned one point, with a total score ranging from 0 to 3 points. A higher qSOFA score has been associated with an increased risk of poor outcomes in sepsis patients, making it a valuable tool for early identification and intervention.

### 2.4. Treatment

Adhering to the principle of “early diagnosis, early rest, early treatment, and on-site treatment”, the comprehensive general treatment is the primary strategy. Early initiation of antiviral treatment with ribavirin (800–1200 mg/d for 5–7 days) was applied in the initial stage [9]. Subsequently, in the middle and late stages, tailored symptomatic treatment was given based on pathological and physiological changes. It included targeted management of fever, chills, and pain; maintaining fluids and electrolytes in balance, blood pressure, and oxygenation based on precise monitoring of hemodynamics invasively or non-invasively; platelet infusion for severe or critical cases with thrombocytopenia and evident bleeding tendency; and CRRT for severe and critically ill patients. Nevertheless, intermittent hemodialysis (IHD) was preferable for AKI patients with stable hemodynamics and fewer potentially fatal complications. Close monitoring and prevention measures of secondary infections, MODS, acute respiratory distress syndrome (ARDS), pulmonary edema, and toxic encephalopathy were also adopted in severe and critical forms.

### 2.5. Data Extraction and Follow-Up

The clinical manifestations of HFRS were closely monitored and recorded during treatment. Whole blood cell count, serum liver enzyme, bilirubin, renal function, and serum electrolyte levels were measured every 2 to 3 days. Clinical data, laboratory findings, and medication information were retrospectively collected. A 30-day prognosis was recorded for each patient. For patients who had been hospitalized for less than 30 days, the 30-day prognosis was obtained through phone interviews.

### 2.6. Laboratory Tests and Examinations

Serum-specific IgM and IgG antibodies were detected using the colloidal gold hantavirus antibody detection kit (Xiamen Bosen Biotech Co., Ltd., Xiamen, China). An initial set of laboratory tests included complete blood count, urine analysis, liver function, renal function, electrolyte levels, coagulation profile, and D-dimer levels. Additionally, HIV antibodies, tumor markers, autoimmune antibodies, and β-human chorionic gonadotropin (β-HCG) were tested. Imaging examinations included abdominal ultrasound (liver, gallbladder, spleen, kidney, ureters, and urinary bladder), chest X-ray, or computed tomography (CT) scans, as well as electrocardiography (ECG), which were performed for all enrolled patients.

### 2.7. Statistical Analysis

Statistical analysis was performed using statistical software R version 4.2.3 (R Core Team, Vienna, Austria). The total patients were randomly divided into the training and validation cohorts in a ratio of 7:3. Continuous variables were expressed as median and interquartile range (median [IQR]). Categorical variables were reported as frequencies and percentages. The continuous variables were compared using the Mann–Whitney U and Kruskal–Wallis tests. The categorical variables were compared using the Pearson Chi-square test. A two-sided *p*-value less than 0.05 was considered statistically significant. To construct a prediction model, each continuous laboratory indicator was further converted into categorical variables based on the cutoff values, which were determined using optimal cutoff analysis with X-tile [10] software based on 30-day mortality. Univariate and multivariate stepwise logistic regression were performed to evaluate independent mortality-related factors. The final multivariable model derived from the training cohort was utilized to develop a nomogram. A risk stratification system was generated using the nomogram based on the total scores of each patient. The maximum total score of the nomogram was divided into the following proportions from low to high risk of death: below 20% as low risk, between 20% and 50% as medium risk, and above 50% as high risk. The Kaplan–Meier survival curve was used to analyze 30-day survival for this new prognostic risk classification system. The diagnostic ability of this nomogram was evaluated by the AUC-PR (precision–recall) curve and the AUC-ROC (receiver operating characteristic) curve. Clinical usefulness was assessed using the calibration curve and decision curve analysis (DCA). Finally, an internal validation to evaluate predictive accuracy was performed with the bootstrap method (1000 bootstrap replicates).

## 3. Results

### 3.1. Epidemiological Characteristics

Our study cohort included 551 subjects after 47 were excluded based on exclusion criteria. HFRS in the studied region exhibits a distinct pattern of monthly seasonal hantavirus infections, with a significant peak from April to July and a minor peak from November to February of the subsequent year (Supplementary Figure S1A). Due to the collection of the studied cases being defined to the years 2016 to 2021, a relative peak of number of hospitalized cases was demonstrated in 2018. The implementation of vaccination programs in 2018 in epidemic areas led to a rapid decrease in the number of hospitalized cases of HFRS in 2019. Furthermore, the ongoing COVID-19 pandemic contributed to a projected decline in patients with HFRS in 2020 and 2021 (Supplementary Figure S1B). Analysis of the age distribution among all cases reveals that individuals aged 51 to 60 accounted for the majority (28.49%,157/551), with males accounting for 71.97% (113/157). The case-mortality rate was 4.17% (23/551) for the overall study population and increased substantially (*p* < 0.001) with age (Supplementary Figure S1C); the case-mortality rates for each age group were 0% (0/34, 19 to 30 years old), 4.26% (2/47, 31 to 40 years), 1.57% (2/127, 41 to 50 years), 3.18% (5/157, 51 to 60 years), 6.11% (8/131, 61 to 70 years), 10.42% (5/48, 71 to 80 years), and 14.29% (1/7, 81 to 86 years), respectively.

### 3.2. Descriptive Analysis

Baseline characteristics, history, complications, and primary treatment details of the HFRS patients randomly divided into training and validation groups in a 7:3 ratio are shown in Table 1. The median age of the patients was 54.0 years, and 70.96% were male. A median of 4 days elapsed between the onset of symptoms and admission. At admission, 73.32% of patients were in fever stage, 6.17% in shock stage, 7.44% in oliguria stage, 8.17% in fever and shock overlap stage, 2.72% in shock and oliguria overlap stage, and 2.18% in fever, shock, and oliguria overlap stage. qSOFA ≥ 2 accounted for 4.72% (26/551), and the one-month mortality rate was 4.17% (23/551). None of the baseline variables showed significant differences between the training and validation cohorts. Supplementary Table S1. provides an overview of the common symptoms and signs of HFRS. Fever (62.07%) emerged as the most prevalent and distinctive presenting symptom, followed by headache (44.83%) and osphyalgia (23.96%). The most frequent hyperemic signs were upper jaw congestion (31.4%), conjunctival congestion (30.49%), and a flushed appearance (12.34%). Bleeding signs were observed across various regions of the body, with the upper chest (1.81%), axilla (1.81%), face (1.63%), and back (1.27%) being commonly affected body parts.

### 3.3. Clinical Index Classification

Using X-tile software (version 3.6), we identified the best cutoff of each peripheral indicator. Subsequently, the continuous laboratory indicators were reclassified into classification indicators based on the cutoff value (Supplementary Table S2).

### 3.4. Univariate and Multivariate Logistic Regression Analysis

A univariate logistic regression analysis was performed on 385 samples of the training cohort to identify variables associated with poor outcomes. Among all the baseline variables, 15 variables were identified as closely related to the unfavorable prognosis (*p* < 0.1) in the univariate analyses. These variables included age, two-stage overlap (fever and hypotensive stage overlap or hypotensive and oliguria stage overlap), three-stage overlap (fever, hypotensive, and oliguria stage overlap), qSOFA ≥ 2, RBC or HGB elevation, thrombocytopenia, WBC or neutrophil elevation, ALT or AST elevation, direct bilirubin elevation, creatinine or uric acid elevation, and hypochloremia. Due to the relatively small sample size of the training cohort, we selected variables with *p* < 0.05 from the univariate analysis to proceed to the next step of multivariate logistic regression analysis. As a result, six risk factors have entered the nomogram, with RBC elevation contributing the most, followed by age, two-stage overlap, qSOFA ≥ 2, AST, and three-stage overlap. Supplementary Table S3 presents the statistical results of both the univariate and multivariate logistic regression analyses. Variables displayed in the baseline table but not shown in Supplementary Table S3 were not found to be independent prognostic factors, including gender, past medical history, complications, treatment, etc.

### 3.5. Nomogram and Risk Classification System for Predicting Prognosis

**Figure 2:**
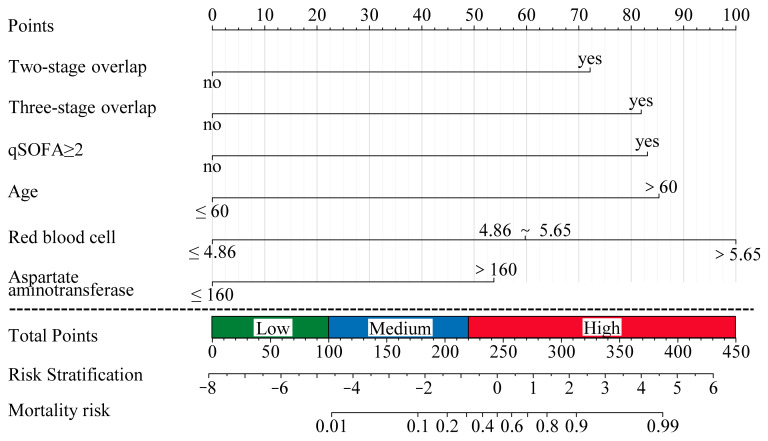
Nomogram (EASTAR) for predicting 30−day mortality of HFRS. Low-risk group (total score ≤ 100), medium-risk group (101–220), and high-risk group (total score ≥ 221).

Furthermore, a nomogram (Figure 2) was established to predict 30-day mortality in HFRS patients based on the independent predictors derived from the results of the multivariate analysis. Each patient’s corresponding values on the respective variable axes were identified using the nomogram, and a vertical line was drawn upward to determine their risk score. These scores were summed, and a vertical line was drawn downward to intersect with the survival axes to determine the probability of 30-day mortality. Furthermore, we constructed a prognostic risk classification system based on the total score calculated via this nomogram chart. Patients in this study had total risk points ranging from 0 to 450. All patients were divided into low-risk group (total score ≤ 100), medium-risk group (101–220), and high-risk group (total score ≥ 221). Kaplan–Meier curves showed that the new risk score system accurately distinguished 30-day survival curves among the three risk groups in the training and validation cohorts (Supplementary Figure S2).

### 3.6. Validation of the Nomogram

**Figure 3:**
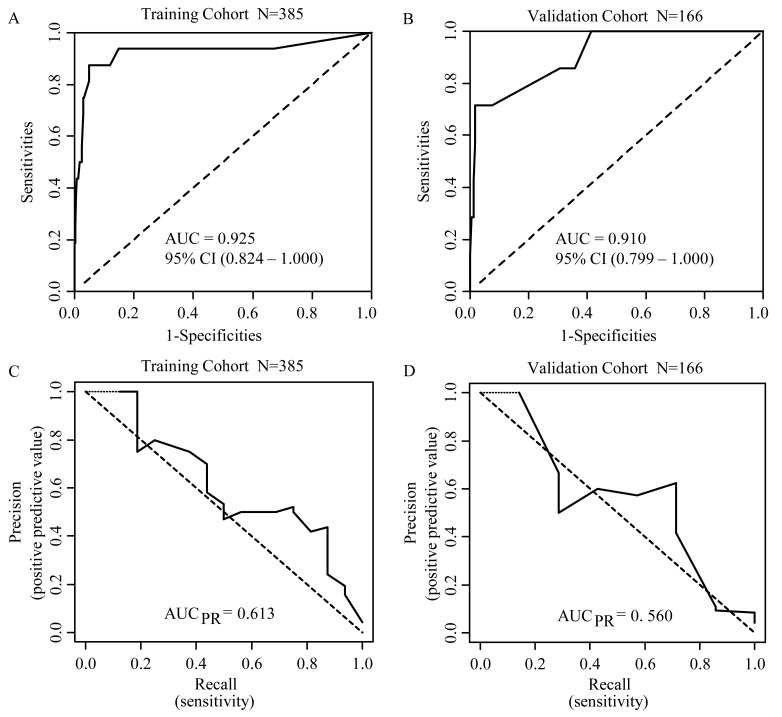
ROC curve and precision–recall (PR) curve analysis of the nomogram for predicting 30−day mortality (**A**) ROC curve analysis in the training cohort and (**B**) in the validation cohort. (**C**) PR curve analysis is in the training cohort, and (**D**) is in the validation cohort.

**Figure 4:**
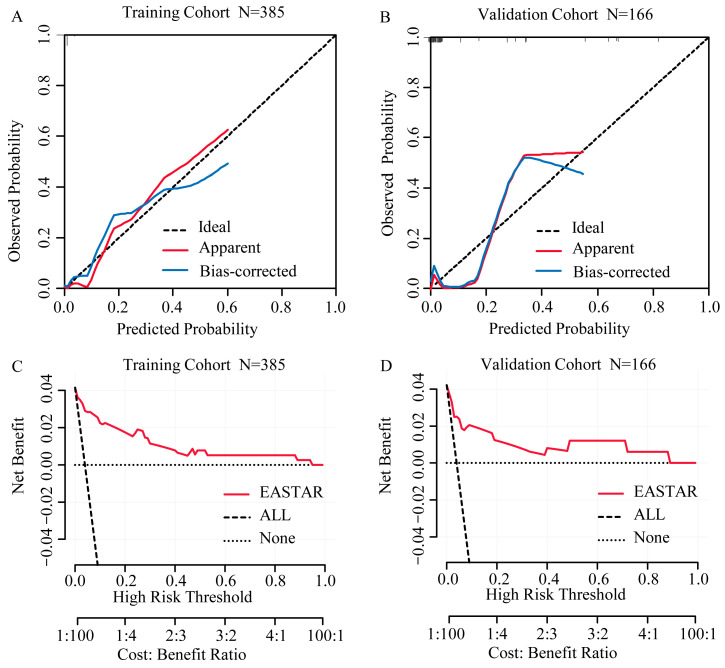
Calibration curve and decision curve analysis (DCA) curve analysis of the nomogram for predicting 30−day mortality (**A**) Calibration curve analysis in the training cohort and (**B**) in the validation cohort. (**C**) DCA curve analysis is in the training cohort, and (**D**) is in the validation cohort.

The area under the receiver operating characteristic (ROC) curve, or the C-index value, serves as a measure of the model’s ability to discriminate between patients who died within 30 days and those who survived. In our study, the AUC for predicting the 30-day mortality of hemorrhagic fever with renal syndrome (HFRS) patients was found to be 0.9249 (95% confidence interval [CI] 0.8237–1.000) in the training cohort and 0.9102 (95% CI 0.7989–1.000) in the validation cohort (Figure 3). These high AUC values indicate a strong predictive accuracy of our model, with the 95% CI suggesting a high level of confidence in the model’s predictive power. The precision–recall curve provides a measure of a model’s ability to balance precision (positive predictive value) and recall (sensitivity). In our analysis, the AUC_PR_ for the training and validation cohorts are 0.613 and 0.560, respectively (Figure 3). These values indicate the trade-off between precision and recall, with higher values suggesting better model performance. Moreover, the calibration curve (Figure 4), which plots the predicted probabilities against the actual outcomes, showed a favorable alignment between the estimated survival probability and the observed survival rate for patients with HFRS. This alignment suggests that our model’s predictions are well calibrated and reliable. Decision curve analysis (DCA) further supports the clinical utility of our nomogram (Figure 4). DCA demonstrated that using our nomogram would lead to a net benefit in predicting 30-day mortality compared to not using the model or using a strategy of treating all or none of the patients. In the internal validation of our prediction model, the bootstrap validation method revealed an accuracy of 0.959, a kappa value of 0.328, and an AUC of ROC of 0.886. The sensitivity of the model was 0.988, indicating a high true positive rate, and the specificity was 0.716, suggesting a reasonable true negative rate. These results underscore the robustness and reliability of our model in predicting 30-day mortality in HFRS patients.

## 4. Discussion

Since 2003, after the implementation of HFRS vaccination in China, the incidence rate of HFRS has significantly decreased [11]. However, it rebounded since 2009 [12]. The inactivated hantaan virus vaccine in South Korea showed a vaccine effectiveness (VE) value of 59.1%, with a higher VE value of 78.7% in high-incidence areas [13]. Since no specific vaccines are approved in Europe or the USA, there are no reports on their impact on prevalence [14]. The overall mortality rate in this up-to-date, largest-scale, multicenter retrospective cohort study was 4.17% (23/551), which is higher than the national average case mortality rate reported in the literature, ranging from 0.54% (2016) to 1.3% (2007) [15]. This disparity can be attributed to the relatively high proportion of severe type (4.44%) and critical type (10.00%) cases with a higher mortality rate, accounting for 45.01% (248/551) and 14.52% (80/551) of the total cases, respectively.

Regarding the annual distribution, there has been a significant decrease in the number of hospitalized cases since reintroducing the hantavirus vaccine to susceptible individuals in epidemic areas started in 2018. The seasonal trends in incidence exhibited a bimodal pattern, with peaks occurring from May to July and November to January. The peak during the summer months is higher, which differs from the nationwide high incidence rate observed during colder months [15]. The seasonal bimodal changes can be attributed to virus type, host reproduction and activity, human behavior, and occupational factors [16]. It is known that among rodents, many species are the main carriers of hantavirus [1,17]. HFRS caused by *Apodemus agrarius* transmission tends to peak during colder months, while Rattus norvegicus transmission peaks during the transition from spring to summer [18]. Notably, Watson et al. reported in 2014 that the Seoul virus (SEOV) primarily resides in *Rattus norvegicus*, whereas the hantaan virus (HTNV) is mainly carried by *Apodemus agrarius* [19]. In a previous report, Hubei Province, where the current study centers are located, was identified as an essential epidemic focus for SEOV [20].

This current study demonstrated that age ≥61 years, two-stage (fever and hypotensive, hypotensive and oliguria) overlap, three-stage (fever, hypotensive, and oliguria) overlap, qSOFA ≥ 2, RBC ≥ 4.87 ×10^12^/L, and AST ≥ 161 IU/L were identified as independent prognostic factors by logistic multivariate analysis and were selected to construct a nomogram to facilitate predictive assessment. These six parameters represent the patient’s baseline condition, stage of disease progression, degree of sepsis, blood volume, and organ damage. Our findings and Fan et al. [21] demonstrate that age is an independent prognostic factor, in contrast to most previous multivariate analyses [5,6,7,22]. Similar to other viral hemorrhagic fever diseases such as Ebola virus disease [23], Lassa fever [24], severe fever with thrombocytopenia syndrome (SFTS) [25], and yellow fever [26], older age has been associated with poorer prognosis. When acute viral hemorrhagic fever diseases occur, the mechanisms by which older age leads to poorer disease prognosis include immunosenescence, inflammaging, telomere shortening, genomic instability, mitochondrial dysfunction, loss of proteostasis, cellular senescence, epigenetic alterations, impaired autophagy, and nutrient sensing dysregulation [27]. These factors contribute to a weakened immune response and increased susceptibility to severe outcomes in elderly individuals.

Hantavirus infection can lead to severe clinical symptoms in individuals with compromised immune status, and complications include tissue edema, bleeding, and kidney injury. Endothelial cells infected with hantavirus result in three significant consequences. First, the loss of endothelial barrier integrity increases vascular permeability, leading to edema. Second, coagulation is activated, initiating platelet aggregation, coagulation dysfunction, and bleeding. Third, cytokine release triggers immune response, instigates inflammation, and maintains the damaged endothelial permeability [28]. The overlap of two or three stages suggests a continuous release of inflammatory factors or, in more severe cases, a cytokine storm, leading to a prolonged fever period. The persistent increase in vascular permeability results in excessive blood volume reduction, and severe endothelial damage causes the rapid onset of severe kidney injury. Consequently, non-hemorrhagic blood volume reduction results in an increase in RBC. Therefore, two-stage or three-stage overlap and elevated RBC are both signs of the critical status of the disease. HFRS, marked by hypovolemic hypotension and AKI, is not ideally assessed with the complex, multi-organ SOFA score [29]. The SOFA score, requiring extensive lab tests and clinical assessments, is complex and time-consuming, hindering rapid emergency evaluations. Conversely, the qSOFA score, comprising just three easy-to-assess indicators—increased respiratory rate, low systolic blood pressure, and altered mental status—is ideal for quick emergency screenings [30]. As one of the parameters for liver dysfunction, increases in AST levels occur in situations of myocardial damage, renal dysfunction, and skeletal muscle damage [31]. An early elevation of AST may occur in patients with hemorrhagic fever due to muscle damage caused by faster capillary damage. As such, it can be an independent early indicator for predicting disease prognosis. Different from the findings of a recent single-center study [32], aiming to establish a predictive model for disease progression assessment, in which six independent risk factors were identified. These include history of hypertension, hypotension upon admission, hypoxemia, neutrophil count, AST, and APTT, where only one parameter, AST, is in common with our EASTAR scoring. The possible explanation could be differences in statistical analyses. Instead of using multivariate logistic regression analysis, an ROC curve analysis was applied for every single parameter in their report.

In this study, HGB, PLT, WBC, neutrophil, ALT, Dbil, and blood chloride were identified as factors influencing prognosis in the univariate analysis but did not retain statistical significance in the multivariate analysis. In the univariate analysis, gender, Tbil, sCr, UA, and blood potassium did not meet the significance threshold of *p* < 0.05 and therefore should not be considered as factors influencing prognosis. Males are more susceptible to hantaviruses due to their engagement in outdoor work like farming, fishing, and construction, which increases their exposure to rodents, the reservoir hosts, consistent with a higher global incidence rate among males, as reported in studies [4,33]. Previous studies [34] have indicated that the severity of thrombocytopenia and leukocytosis is an independent risk factor for severe AKI in HFRS. However, these studies [34] selected the lowest PLT and highest WBC during the disease course, which differs from our study that focused on the baseline PLT and WBC levels at admission. It should be noted that the levels of PLT may be affected by platelet-stimulating drugs such as thrombopoietin (TPO) and platelet transfusions, and secondary bacterial infections can increase WBC levels. Nevertheless, multivariate analysis revealed that ribavirin, blood purification therapy, and platelet transfusion were not associated with disease prognosis. Although a clinical study involving 242 patients reported that ribavirin treatment reduced the mortality rate of HFRS [35], recent studies have not found ribavirin effective in treating hantavirus infections [9].

In clinical practice, a nomogram is a valuable tool with a user-friendly graphical interface for quickly and rapidly predicting specific variables [5]. However, two previous studies are single-center retrospective with relatively small sample sizes. Li’s study focuses on pediatric cases of relatively moderate illness, limiting its application to adult patients [6]. On the other hand, Yang’s research did not collect longitudinal data over a continuous period [7]. In addition, other prognosis prediction studies for HFRS either lack available prediction models [22] or rely on a single laboratory indicator, such as platelet distribution width (PDW) [21], procalcitonin (PCT) [36], or serum adenosine deaminase (ADA) [37]. Due to variations in clinical stages of HFRS and the impact of decreased kidney excretion function on blood indicators during AKI, it is difficult to accurately evaluate the severity and predict clinical outcomes with a single laboratory test parameter. We also developed a prognostic risk score system based on our nomogram and categorized patients into low, medium, and high risks. We name this risk scoring system EASTAR (**E**rythrocyte, **A**ge, q**S**OFA scores, overlap of **T**wo or three stages, and elevated **A**ST, **R**isk score system). The EASTAR is an effective triage tool for identifying the urgent admission of high-risk patients to the ICU. The AUC of ROC analysis to test model differentiation was 0.9102 in the validation cohort. Despite this, ROC analysis is unaffected by our imbalanced data and cannot fully evaluate the model’s performance [38]. A precision–recall curve analysis was performed, and the result was 0.56 in the validation cohort. Calibration curves were employed to assess the nomogram, and the optimal agreement between the nomogram’s predicted probabilities and the observed values indicates a high level of goodness of fit. Moreover, we performed a decision curve analysis (DCA) to clarify the clinical usefulness of our risk-stratification model. Internal validation using bootstrap resampling methods showed our model has optimism, suggesting an excellent capacity to predict 30-day mortality in HFRS. Specifically, the high AUC value from the ROC analysis and the precision–recall curve analysis result underscore the model’s effectiveness in distinguishing between patients who require ICU admission. Furthermore, the decision curve analysis clarifies the clinical usefulness of our risk-stratification model, demonstrating its potential to improve patient outcomes through better risk classification. These findings collectively demonstrate that the EASTAR system is highly effective in predicting outcomes and identifying patients who need ICU care. As a user-friendly tool for assessing HFRS severity, the EASTAR system aids in precise management and ultimately improves patient outcomes.

The present study possesses several limitations. Firstly, although it is a multicenter study, the population is predominantly concentrated in central China, primarily Hubei Province, limiting its generalizability to a broader population. External validation is needed in HFRS patients from other regions to demonstrate the generalizability and predictive performance. Secondly, the diagnosis of HFRS in this retrospective study depended on patients’ medical history, clinical manifestations, and serological tests without RT-PCR diagnosis and virus sequencing, lacking data on virus load and virus typing. Additionally, due to the study’s retrospective nature, specific crucial inflammation and immune indicators were missing, resulting in the suboptimal predictive performance of the nomogram. To address these limitations, future prospective research with comprehensive clinical indicators should be conducted to explore the predictive ability of immune indicators and further analyze virus subtypes and even genomic differences.

## 5. Conclusions

Our study revealed several key findings. In central China, HFRS displayed a dual seasonal pattern, but the peak was higher in the summer than in other regions. RBC ≥ 4.87 ×10^12^/L, age ≥ 61 years, qSOFA ≥ 2, two-stage (fever and hypotensive, hypotensive and oliguria) overlap, three-stage (fever, hypotensive, and oliguria) overlap, and AST ≥ 161 IU/L were independent risk factors for HFRS patients. Based on this nomogram derived from the above six indicators, a risk score system (EASTAR) was constructed and proved effective in predicting prognosis. By using this score, clinicians can quickly stratify the risk of HFRS patients and allow high-risk patients to be admitted to the ICU for intensive life support. Further validation of this prognostic risk score will be conducted in a prospective multicenter study.

## Figures and Tables

**Table 1:** Baseline Characteristics, complications, and primary treatment details of HFRS patients. Data are n (%) or median (IQR). Databases have no missing values. Cohorts were randomly divided in a 7:3 ratio. *p* values describe the comparison between training and validation cohort. Interval, days from symptom onset to hospital admission; qSOFA, quick Sequential Organ Failure Assessment; RBC, red blood cell; HGB, hemoglobin; PLT, platelet; WBC, white blood cell; ARDS, acute respiratory distress syndrome; MODS, multiple organ dysfunction syndrome; CRRT, continuous renal replacement therapy.

Characteristics	Total (N = 551)	Training (N = 385)	Validation (N = 166)	*p* Value
Age, years	54.00 [46.50;64.00]	55.00 [47.00;64.00]	53.00 [45.25;63.75]	0.311
gender: male	391 (70.96%)	267 (69.35%)	124 (74.70%)	0.243
female	160 (29.04%)	118 (30.65%)	42 (25.30%)	
Interval, days	4.00 [3.00;6.00]	4.00 [3.00;6.00]	4.00 [3.00;6.00]	0.543
Deceased	23 (4.17%)	16 (4.16%)	7 (4.22%)	1
qSOFA ≥ 2	26 (4.72%)	17 (4.42%)	9 (5.42%)	0.77
RBC, ×10^12^/L	4.37 [3.87;5.08]	4.36 [3.86;5.10]	4.40 [3.96;5.03]	0.698
HGB, g/L	138.00 [121.00;158.00]	137.00 [120.00;158.00]	138.50 [122.25;156.75]	0.707
PLT, ×10^9^/L	45.00 [27.00;81.00]	45.00 [27.00;80.00]	50.00 [27.00;81.75]	0.561
WBC, ×10^9^/L	10.80 [6.70;16.80]	11.00 [6.80;17.80]	10.07 [6.12;15.52]	0.109
Neutrophil, ×10^9^/L	6.56 [4.22;11.14]	6.69 [4.33;11.23]	6.13 [4.06;10.48]	0.103
Lymphocyte, ×10^9^/L	1.95 [0.92;3.72]	2.10 [0.99;3.85]	1.71 [0.84;3.34]	0.079
Allotypic lymphocyte	304 (55.17%)	218 (56.62%)	86 (51.81%)	0.342
Alanine aminotransferase, IU/L	44.00 [28.00;69.00]	42.00 [27.00;67.00]	47.50 [29.25;72.75]	0.104
Aspartate amino-transferase, IU/L	80.00 [47.00;133.50]	78.00 [46.00;132.00]	82.50 [50.25;142.00]	0.442
Total bilirubin, μmol/L	11.20 [8.10;15.85]	10.90 [8.00;15.80]	11.40 [8.40;15.95]	0.242
Direct bilirubin, μmol/L	3.90 [2.80;5.60]	3.80 [2.80;5.50]	4.10 [2.80;5.80]	0.34
Creatinine, μmol/L	131.00 [83.75;271.05]	132.00 [85.00;254.00]	130.50 [81.00;299.00]	0.658
Blood urea nitrogen, mmol/L	10.77 [6.33;17.43]	10.67 [6.46;16.99]	10.98 [5.94;18.15]	0.869
Uric acid, μmol/L	369.00 [268.00;511.30]	371.00 [271.00;492.00]	362.50 [264.75;539.98]	0.699
Potassium, mmol/L	3.83 [3.51;4.20]	3.83 [3.50;4.21]	3.82 [3.54;4.14]	0.694
Sodium, mmol/L	136.20 [132.60;139.30]	136.00 [132.80;139.20]	136.40 [132.60;139.83]	0.369
Chlorine, mmol/L	96.90 [94.35;100.80]	97.60 [94.50;101.00]	96.40 [94.10;99.88]	0.101
Calcium, mmol/L	2.00 [1.87;2.11]	1.99 [1.87;2.10]	2.02 [1.89;2.13]	0.235
Urinary protein	357 (64.79%)	255 (66.23%)	102 (61.45%)	0.326
Hematuria	185 (33.58%)	135 (35.06%)	50 (30.12%)	0.303
Clinical stage:				0.589
Fever stage	404 (73.32%)	282 (73.25%)	122 (73.49%)	
Shock stage	34 (6.17%)	27 (7.01%)	7 (4.22%)	
Oliguria stage	41 (7.44%)	27 (7.01%)	14 (8.43%)	
Fever and shock overlap	45 (8.17%)	28 (7.27%)	17 (10.24%)	
Shock and oliguria overlap	15 (2.72%)	12 (3.12%)	3 (1.81%)	
Fever, shock, and oliguria overlap	12 (2.18%)	9 (2.34%)	3 (1.81%)	
History				
Type 2 diabetes	33 (5.99%)	24 (6.23%)	9 (5.42%)	0.863
Hypertension	60 (10.89%)	40 (10.39%)	20 (12.05%)	0.671
Chronic hepatitis B	43 (7.80%)	29 (7.53%)	14 (8.43%)	0.85
Nephrolithiasis	78 (14.16%)	55 (14.29%)	23 (13.86%)	1
Hepatic fibrosis in schistosomiasis	60 (10.89%)	44 (11.43%)	16 (9.64%)	0.638
Hyperthyroidism and Hyperthyroidism	32 (5.81%)	20 (5.19%)	12 (7.23%)	0.46
Complications				
Ascites	80 (14.52%)	60 (15.58%)	20 (12.05%)	0.342
Pleural effusion	135 (24.50%)	98 (25.45%)	37 (22.29%)	0.494
Pulmonary infection	184 (33.39%)	132 (34.29%)	52 (31.33%)	0.564
Pancreatitis	49 (8.89%)	40 (10.39%)	9 (5.42%)	0.086
Gastrointestinal bleeding	4 (0.73%)	3 (0.78%)	1 (0.60%)	1
Respiratory bleeding	1 (0.18%)	1 (0.26%)	0 (0.00%)	1
Urogenital bleeding	1 (0.18%)	1 (0.26%)	0 (0.00%)	1
ARDS	2 (0.36%)	1 (0.26%)	1 (0.60%)	0.512
Pulmonary edema	2 (0.36%)	1 (0.26%)	1 (0.60%)	0.512
Cerebral edema	1 (0.18%)	0 (0.00%)	1 (0.60%)	0.301
MODS	6 (1.09%)	6 (1.56%)	0 (0.00%)	0.185
Treatment				
Ribavirin	443 (80.40%)	307 (79.74%)	136 (81.93%)	0.634
Platelet infusion	78 (14.16%)	54 (14.03%)	24 (14.46%)	1
Hemodialysis	27 (4.90%)	17 (4.42%)	10 (6.02%)	0.557
CRRT	83 (15.06%)	64 (16.62%)	19 (11.45%)	0.153
Oxygen therapy	418 (75.86%)	293 (76.10%)	125 (75.30%)	0.925

## Data Availability

All data are shown in the manuscript.

## Supplementary Materials

The following supporting information can be downloaded at: https://www.mdpi.com/article/10.3390/tropicalmed10020051/s1, Figure S1: Epidemiological characteristics of HFRS (A) Seasonal characteristics of Hantavirus infection; (B) Incidence by year of Hantavirus infection from 2016 to 2021; (C) Case mortality rate at different age ranges; Figure S2: Kaplan-Meier 30−day survival curves of HFRS patients at different risks stratified by the scoring of the nomogram (A) in the training cohort. (B) in the validation cohort; Table S1: The common symptoms and signs of HFRS patients; Table S2: The continuous laboratory indicators were reclassified into classification indicators based on the cutoff value; Table S3: Univariate and multivariate logistic regression analysis of risk factors for mortality in patients with HFRS.

## Abbreviations

HFRS	Hemorrhagic fever with renal syndrome
qSOFA	quick Sequential Organ Failure Assessment
RBC	Red blood cell count
AST	Aspartate aminotransferase
NE	Nephropathiaepidemica
HCPS	Hantavirus cardiopulmonary syndrome
AKI	Acute kidney injury
MODS	Multiple organ dysfunction syndrome
ARDS	Acute respiratory distress syndrome
CRRT	Continuous renal replacement therapy
ICU	Intensive care unite
ICD-10	International Classification of Diseases 10
MELD	Model for End-Stage Liver Disease
PDW	Platelet distribution width
PCT	Procalcitonin
ADA	Serum adenosine deaminase
IgM	Immunoglobulin M
IgG	Immunoglobulin G
MAP	Mean arterial pressure
IHD	Intermittent hemodialysis
β-HCG	β-human chorionic gonadotropin
CT	Computed tomography
ECG	Electrocardiography
SFTS	Severe fever with thrombocytopenia syndrome
HGB	hemoglobin
PLT	Platelet count
WBC	White blood cell count
ALT	Alanine aminotransferase
Tbil	Total bilirubin
Dbil	Direct bilirubin
sCr	Serum creatinine
UA	Uric acid
TPO	Thrombopoietin
ROC	Receiver operating characteristic curve
