# Supplementary material for: Clinical Characteristics and a Novel Prediction Nomogram (EASTAR) for Patients with Hemorrhagic Fever with Renal Syndrome: A Multicenter Retrospective Study
Source: Trop Med Infect Dis. 2025 Feb 8;10(2):51. doi: 10.3390/tropicalmed10020051 (PMC11860278; doi:10.3390/tropicalmed10020051)
Supplement: Supplementary file 1 [file tropicalmed-10-00051-s001.zip › tropicalmed-3299527-supplementary.pdf]

Supplementary Materials

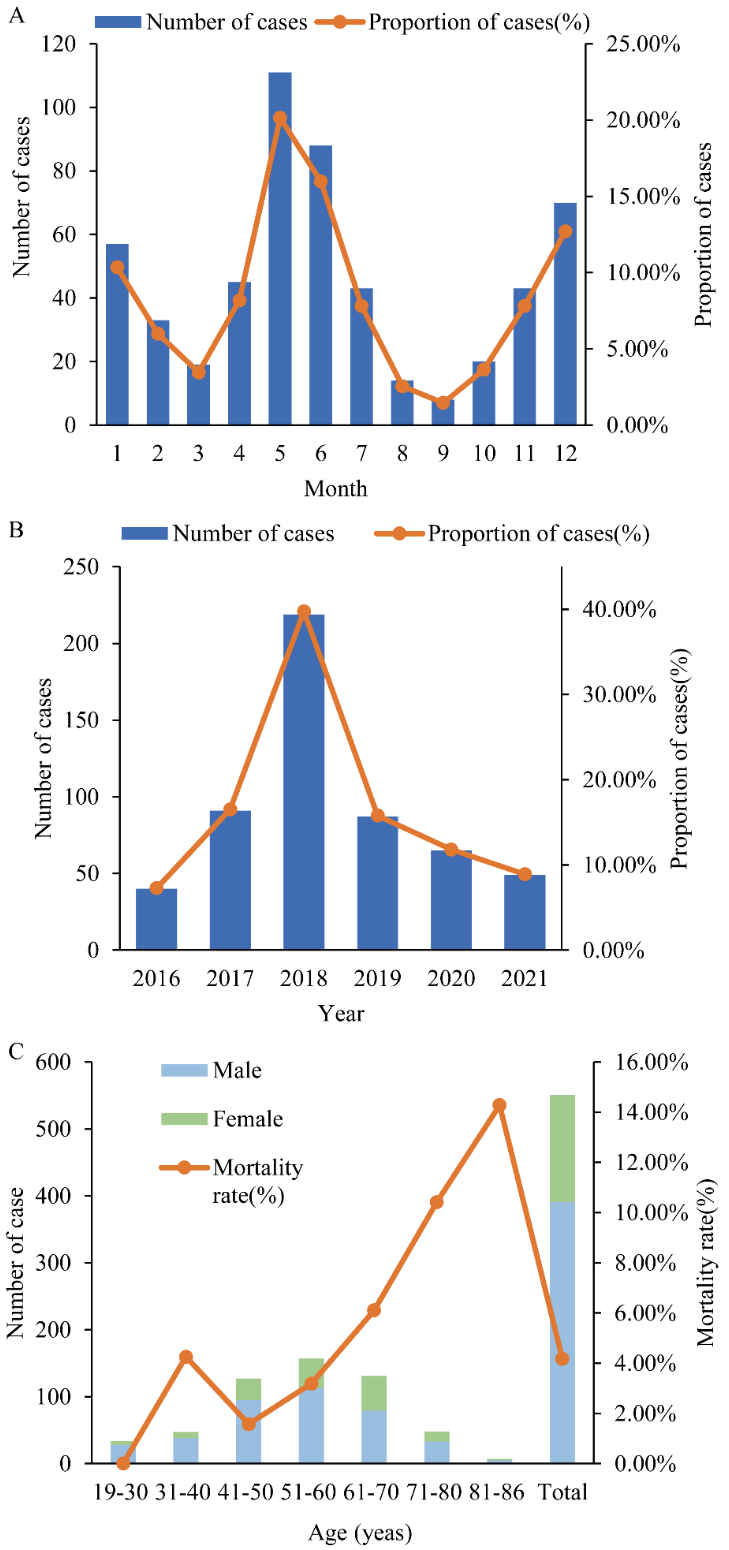

Supplementary Figure S1. Epidemiological characteristics of HFRS (A) Seasonal characteristics of Hantavirus infection; (B) Incidence by year of Hantavirus infection

from 2016 to 2021; (C) Case mortality rate at different age ranges.

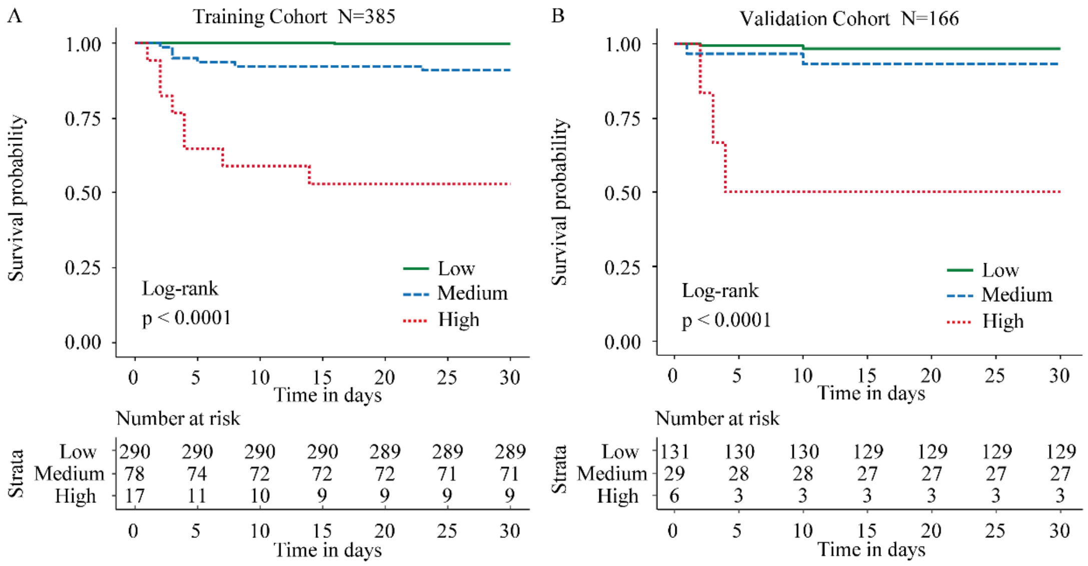

Supplementary Figure S2. Kaplan-Meier 30−day survival curves of HFRS patients at different risks stratified by the scoring of the nomogram (A) in the training cohort. (B) in the validation cohort.

Supplementary Table S1. The common symptoms and signs of HFRS patients.

**Symptoms**		**Number of cases**	**Incidence ratio (%)**	**Hyperemic signs**	**Number of cases**	**Incidence ratio (%)**
Fever		342	62.07%	Upper jaw congestion	173	31.40%
Shivering		23	4.17%	Conjunctival congestion	168	30.49%
Pain	Headache	247	44.83%	Flushed appearance	68	12.34%

	Osphyalgia	132	23.96%			
	Orbital pain	54	9.80%	Bleeding signs		
	Myalgia	29	5.26%	Forebreast	10	1.81%
	Arthralgia	12	2.18%	Axilla	10	1.81%
Digestive tract	Anorexia	406	73.68%	Face	9	1.63%
	Nausea	299	54.26%	Back	7	1.27%
	Vomit	185	33.58%	All over the body	6	1.09%
	Diarrhea	156	28.31%	Neck	6	1.09%
	Abdominal pain	80	14.52%	Shoulder	5	0.91%
	Abdominal distension	38	6.90%	Lip	2	0.36%
Respiratory tract	Cough	62	11.25%	Wrist	1	0.18%
Central nervous system	Delirium	1	0.18%			

Supplementary Table S2. The continuous laboratory indicators were reclassified into classification indicators based on the cutoff value. In training cohort, clinical indicators were graded according to 30−day mortality by using the X-tile software. P values describe the 30−day Kaplan-Meier survival curve analysis of subgroup analyses grouped by clinical grade. qSOFA, quick Sequential Organ Failure Assessment.

Characteristics	Clinical grade			P value
	1	2	3	
qSOFA≥2	≤1	≥2		< 0.0001
Age, years	≤60	≥61		0.0200
Red blood cell, $\times 10^{12}$/L	≤4.86	4.87~5.64	≥5.65	0.0034
Hemoglobin, g/L	≤142	143~174	≥175	0.0064
Platelet, $\times 10^{9}$/L	≤37	≥38		0.0004
White blood cell, $\times 10^{9}$/L	≤11	11.01~25.69	≥25.7	0.0200
Neutrophil, $\times 10^{9}$/L	≤7.79	7.80~17.79	≥17.8	0.0086
Lymphocyte, $\times 10^{9}$/L	≤3.00	≥3.01		0.1900
Alanine aminotransferase, IU/L	≤90	≥91		< 0.0001
Aspartate amino-transferase, IU/L	≤160	≥161		< 0.0001
Total bilirubin, μmol/L	≤13.89	≥13.9		0.1500
Direct bilirubin, μmol/L	≤6.49	≥6.5		0.0250
Creatinine, μmol/L	≤108.00	≥108.01		0.1900
Blood urea nitrogen, mmol/L	≤8.21	≥8.22		0.1900
Uric acid, μmol/L	≤410.00	≥410.01		0.8200
Potassium, mmol/L	≤4.25	≥4.26		0.1100

Sodium, mmol/L	≤132.10	≥132.11	0.4400
Chlorine, mmol/L	≤93.99	≥94.00	0.1700
Calcium, mmol/L	≤2.03	≥2.04	0.7400

Supplementary Table S3. Univariate and multivariate logistic regression analysis of risk factors for mortality in patients with HFRS.

	Univariate logistic regression analysis				Multivariate logistic regression analysis			
	Estimate	Std. Error	z value	P value	Estimate	Std. Error	z value	P value
Two-stage overlap△	1.7769	0.5469	3.249	0.0012	2.3299	0.7618	3.06	0.0022
Three-stage overlap○	2.6363	0.7614	3.463	0.0005	2.6455	1.1261	2.35	0.0188
gender	0.3198	0.5287	0.605	0.5450				
qSOFA≥2	2.9718	0.6003	4.95	< 0.0001	2.6834	0.8828	3.04	0.0024
Age	1.1797	0.5280	2.234	0.0255	2.7550	0.8473	3.25	0.0011
Red blood cell	2.7328	0.7111	3.843	0.0001	3.2294	0.9591	3.37	0.0008
Hemoglobin	2.8317	0.8352	3.390	0.0007				
Platelet	-1.6022	0.5872	-2.729	0.0064				
White blood cell	1.5540	0.6677	2.327	0.0199				
Neutrophil	1.7554	0.6768	2.594	0.0095				

Alanine aminotransferase	2.3185	0.5376	4.313	< 0.0001				
Aspartate amino-transferase	2.3882	0.5570	4.287	< 0.0001	1.7364	0.7219	2.41	0.0162
Total bilirubin	0.8178	0.5126	1.595	0.1110				
Direct bilirubin	1.3679	0.5231	2.615	0.0089				
Creatinine	1.1327	0.6491	1.745	0.0810				
Uric acid	0.8893	0.5272	1.687	0.0916				
Potassium	0.7577	0.5315	1.426	0.1540				
Chlorine	-1.3661	0.5165	-2.645	0.0082				

△Two-stage overlap: fever and hypotensive stage overlap or hypotensive and oliguria stage overlap

○Three-stage overlap: fever, hypotensive, and oliguria stage overlap

Std. Error, standard error; z value, Chi-square value; qSOFA, quick Sequential Organ Failure Assessment.
